# Defining Breadth of Hepatitis C Virus Neutralization

**DOI:** 10.3389/fimmu.2018.01703

**Published:** 2018-08-02

**Authors:** Valerie J. Kinchen, Justin R. Bailey

**Affiliations:** Department of Medicine, Johns Hopkins University School of Medicine, Baltimore, MD, United States

**Keywords:** hepatitis C virus, Flaviviridae, antibodies, neutralizing breadth, viral diversity

## Abstract

Extraordinary genetic diversity is a hallmark of hepatitis C virus (HCV). Therefore, accurate measurement of the breadth of antibody neutralizing activity across diverse HCV isolates is key to defining correlates of immune protection against the virus, and essential to guide vaccine development. Panels of HCV pseudoparticle (HCVpp) or replication-competent cell culture viruses (HCVcc) can be used to measure neutralizing breadth of antibodies. These *in vitro* assays have been used to define neutralizing breadth of antibodies in serum, to characterize broadly neutralizing monoclonal antibodies, and to identify mechanisms of HCV resistance to antibody neutralization. Recently, larger and more diverse panels of both HCVpp and HCVcc have been described that better represent the diversity of circulating HCV strains, but further work is needed to expand and standardize these neutralization panels.

## Hepatitis C Virus (HCV) Viral Diversity and the Neutralizing Antibody Response

**Figure 1:**
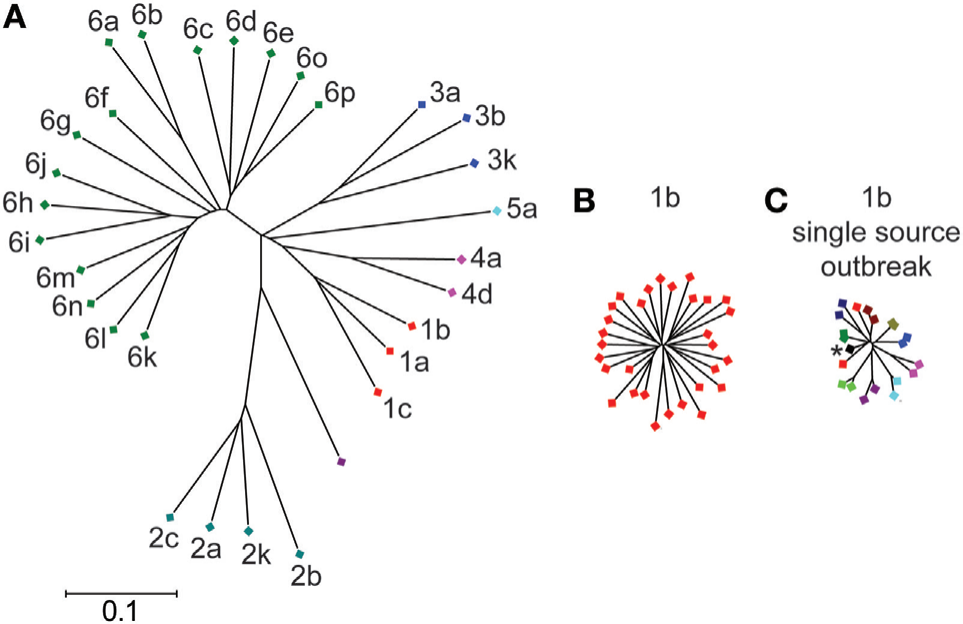
Genetic diversity of hepatitis C virus (HCV) E1E2 across subtypes and within a single subtype. Phylogenetic trees of E1E2 amino acid sequences. E1E2 amino acid sequences from multiple subtypes **(A)** or a single subtype **(B)** were downloaded from the LANL HCV sequence database. Not all known subtypes are shown. **(C)** E1E2 amino acid sequences of 10 individuals from the Irish anti-D cohort, who were all infected from the same inoculum (asterisk) between 1977 and 1978 (9). Sequences of virus from each individual are indicated with a different color. Trees were inferred using the Neighbor-joining method, with branch lengths drawn to scale. Subtypes are labeled. All three trees are on the same scale. Distances were computed using the JTT method. Analyses were performed in MEGA7 (10).

Interferon-free therapies for HCV have revolutionized treatment of those infected with the virus, but a vaccine to prevent infection is needed (1). The extensive genetic diversity of HCV has been a major obstacle to vaccine development. HCV is genetically heterogeneous with seven genotypes and more than 80 subtypes (Figure 1). Within the envelope genes (E1 and E2), approximately 30% of amino acids differ between strains from different genotypes, while strains from different subtypes within each genotype differ at approximately 20% of their E1E2 amino acids (2–5). Even viral strains within the same subtype differ at up to 10% of their E1E2 amino acids. Within an infected individual, error-prone replication by HCV and immune selection lead to the generation of a viral swarm made up of many distinct strains, providing opportunities for expansion of antibody resistant variants (6–8). Therefore, induction of high-titer cross reactive antibodies that are capable of neutralizing diverse viruses within subtypes and across multiple genotypes may be required for an effective vaccine.

Significant effort has been devoted to development of vaccines intended to induce protective antibodies against HCV, but most vaccines tested to date in nonhuman primates or humans have induced humoral responses with relatively poor activity against heterologous HCV strains [reviewed in Ref. (11, 12)]. For example, a vaccine composed of recombinant full-length E1E2 protein from a single HCV strain has been tested in both chimpanzees and humans. Vaccination was protective against homologous HCV challenge in chimpanzees (12). However, the same vaccine reduced rates of persistence but did not prevent infection in chimpanzees after challenge with a heterologous virus (13), and neutralizing antibodies against heterologous HCV strains were induced in only three of 16 vaccines in a phase 1a human trial (14). Overall, these and other vaccine trials suggest that induction of antibodies with broad neutralizing activity across diverse HCV strains may be a critical challenge for HCV vaccine development.

## Neutralizing Antibody Mediated Protection Against HCV Infection

Antibodies capable of neutralizing HCV infection (NAbs) target the viral envelope glycoproteins, E1 and E2, which are membrane-anchored proteins believed to form a heterodimer on the surface of viral particles. E2 interacts with multiple cell surface receptors, including but not limited to CD81 and scavenger receptor B1 (SR-B1), while the function of E1 remains unclear (15). Most HCV-infected individuals develop strain-specific NAbs against hypervariable region 1 (HVR1), a 27-amino acid region at the amino-terminus of E2, but viral escape mutations generally confer resistance to these antibodies (6, 16–20). In contrast, NAbs capable of neutralizing infection by multiple diverse HCV strains *in vitro*, commonly called broadly neutralizing antibodies (bNAbs), develop in some infected individuals. These bNAbs generally bind to relatively conserved epitopes at the CD81 receptor binding site of E2 (CD81bs) or to the E1E2 heterodimer [reviewed in Ref. (21, 22)].

There is evidence from studies of HCV infection of humans and animal models that bNAbs can be protective. Notably, early-developing HCV-specific bNAb responses are associated with clearance of primary human HCV infection (23–26). Pestka et al. demonstrated that women infected during a single-source outbreak of HCV were more likely to clear the virus if they developed serum antibodies early in infection that were capable of neutralizing at least one heterologous HCV strain *in vitro*. Similarly, Osburn et al. demonstrated that plasma isolated immediately prior to clearance of HCV infection neutralized a median of 6 of 19 heterologous HCV strains, while acute infection plasma of control subjects with subsequent persistence of infection neutralized a median of only 1 of 19 heterologous strains. In addition, individuals who clear one infection clear subsequent infections more than 80% of the time, and clearance of reinfection is associated with rapid induction of antibodies capable of neutralizing heterologous HCV strains *in vitro* (27).

There is also evidence of protection in animal models of HCV infection. Infusion of immunoglobulin isolated from the serum of a chronically infected human prior to challenge with homologous virus from the same donor prevented infection of most human liver chimeric mice. Infusion of this chronic-phase human immunoglobulin prior to challenge of a chimpanzee prevented infection with homologous, but not heterologous HCV strains (28–30). In contrast, infusion of monoclonal bNAbs prior to challenge with heterologous viruses prevented infection in most human liver chimeric mice (31–33) and chimpanzees (34), and combinations of monoclonal bNAbs also abrogated established HCV infection in a human liver chimeric mice (35). Together, these studies demonstrate that induction of bNAbs may be necessary to prevent infection by diverse, heterologous HCV strains.

## Experimental Systems for Quantitation of Antibody Neutralizing Activity

Assessment of antibody neutralization of HCV relies largely upon two different *in vitro* systems: the HCV pseudoparticle (HCVpp) system and the replication-competent HCV cell culture (HCVcc) system. HCVpp are retroviral particles with HCV envelope glycoproteins (E1 and E2) on their surface. These particles can be produced by transfection of HEK-293T cells with an E1E2-expressing plasmid and a plasmid expressing an envelope-defective HIV-1 genome with a luciferase reporter. Alternatively, cells can be transfected with an E1E2-expressing plasmid, a murine leukemia virus (MLV) Gag/Pol packaging construct, and a luciferase-encoding reporter plasmid. In either case, after transfection, enveloped particles bearing HCV E1E2 proteins on their surface are released through a retroviral budding process into culture supernatant, enabling the measurement of single rounds of viral entry into hepatoma cells or primary human hepatocytes. HCVpps were used to identify many of the cell surface receptors required for HCV entry (36–40). Unlike HCVpp, HCVcc reproduce the full replication cycle of HCV *in vitro* (41–45) and in animal models (46–48). HCVccs are produced through transfection of *in vitro* transcribed full-genomic HCV RNA into hepatoma cells. Relatively few HCV strains are capable of replication in *in vitro*, but recently strains from genotypes 1–3 have been adapted to allow *in vitro* replication (49–53). After initial transfection, culture supernatants are infectious and can be passaged serially *in vitro*. The HCVcc system has been used to further define the viral entry pathway (54–57).

Both HCVpp and HCVcc panels have been developed to express diverse E1E2 strains, including strains from genotypes 1–6 (HCVpp) (58, 59) and genotypes 1–7 (chimeric HCVcc) (42, 60), enabling the assessment of breadth of antibody neutralizing activity. Notably, HCVpps were used in studies described earlier in this review which demonstrated that the early development of bNAbs was associated with clearance of HCV infection (23–26). HCVccs were used in the studies described earlier which demonstrated that antibodies with broad *in vitro* neutralizing activity can prevent HCV infection in animal models (31–34).

The development of diverse panels of HCVpp or HCVcc has been complicated by the relatively high frequency with which primary E1E2 isolates are poorly functional or nonfunctional *in vitro*, producing HCVpp that do not mediate detectable entry into hepatoma cells, or chimeric HCVcc that do not replicate in *in vitro* (61, 62). Recently, efforts have been made to address these technical obstacles. E1E2 clones that are poorly functional when pseudotyped with HIV-1 Gag protein may produce functional HCVpp when pseudotyped with MLV Gag protein, or vice versa. The mechanisms explaining this phenomenon are unknown. In addition to pseudotyping with either HIV-1 or MLV, optimization of ratios of MLV and HCV E1E2 plasmids during transfection can also improve the function of some E1E2 strains in HCVpp (63). Along with technical improvements in HCVpp production, technology for production of HCVcc has also advanced. The development of chimeric HCVcc based on the genotype 2a JFH1 strain with Core-NS2 genes from multiple genotypes has expanded the number of diverse HCVcc available for neutralization studies. These chimeric HCVccs have an advantage relative to some full-length HCVcc strains, since adaptive mutations required for efficient replication *in vitro* are outside of E1E2 in most cases. Therefore, the E1E2 genes expressed accurately represent the sequences of naturally circulating viruses. Two recent studies described cloning of dozens of naturally occurring E1E2 genes into HCVcc chimeras, generating replication-competent viruses (61, 64). Wasilewski et al. used an E1E2-deleted H77/JFH-1 chimeric construct with an introduced restriction site to facilitate high-throughput insertion of diverse E1E2 genes.

As more E1E2-matched HCVpp and HCVcc are produced, some phenotypic differences between E1E2 expressed using the two systems have been identified. For reasons that are still unknown, some E1E2 strains that produce replication-competent HCVcc chimeras produce poorly functional HCVpp, and, conversely, some E1E2 strains that are functional in HCVpp do not produce replication-competent HCVcc chimeras (62). In addition, HCVpp tend to be generally more neutralization sensitive than HCVcc (61, 62), perhaps due to structural differences between HCVcc and HCVpp, including the association of apolipoproteins with HCVcc but not HCVpp (65, 66). Fortunately, despite these differences, multiple studies have demonstrated concordance between the neutralization results of identical E1E2 clones expressed in either HCVpp or HCVcc, including concordance in the rank order of neutralization sensitivity of different E1E2 strains and concordance in resistance phenotypes of specific mutations (61, 62, 64, 67). These results suggest that, for most experiments, either HCVpp or HCVcc can be used to measure antibody neutralizing activity, expanding the diversity of E1E2 isolates available for neutralization assays.

## HCV Panels Used to Quantitate Neutralizing Breadth

Defining the neutralizing breadth of anti-HCV monoclonal antibodies and immune sera is critical to understand the antibody response required to protect against circulating HCV strains. Due to the previously limited availability of diverse E1E2 isolates in HCVcc and HCVpp, the neutralizing breadth of anti-HCV antibodies has traditionally been measured using relatively small panels of HCVcc or HCVpp (31, 36, 42, 59, 68, 69). As described earlier in this review, these smaller panels have greatly advanced understanding of virus neutralization, but they do not express many of the polymorphisms common in natural HCV isolates, and, therefore, they may overestimate neutralizing breadth of antibodies or sera. In addition, many of these studies have measured neutralization of historically important reference strains of HCV (31, 36, 42, 59, 68–70), such as the genotype 1a isolate, H77, which is highly sensitive to neutralization (69).

Recently, panels of HCVpp and HCVcc have been expanded to encompass more of the diversity of circulating HCV strains (Table 1). A panel of HCVpp from the University of Nottingham includes 78 HCVpp predominantly from genotype 1, but also including some isolates from genotypes 2–6 (61). A second panel developed at Johns Hopkins University comprises natural genotype 1a and 1b E1E2 isolates from cohorts in the US and Ireland. This panel includes 113 HCVpp and expresses 97% of amino acid polymorphisms present at greater than 5% frequency in a reference set of 643 genotype 1 HCV isolates submitted to GenBank from around the world (71). Sensitivity of HCVpp in this panel to neutralization by two bNAbs varied by more than 100-fold (67). A 19-HCVpp subset of this panel expresses 94% of amino acid polymorphisms present at greater than 5% frequency in worldwide genotype 1 sequence (72). This 19 HCVpp genotype 1 panel has been used extensively to define neutralizing breadth of mAbs and sera (9, 23, 27, 62, 64, 72, 73). While significantly fewer replication-competent HCVcc strains are available, progress has been made in expanding the number of isolates (61–63, 74). Most recently, Carlsen et al. applied a panel of chimeric HCVcc constructs expressing 16 E1E2 genes from genotypes 1–3. Like the diverse HCVpp in the Nottingham and Hopkins panels, these HCVcc also varied widely in sensitivity to neutralization by monoclonal antibodies (74).

**Table 1:** HCV cell culture (HCVcc) and HCV pseudoparticle (HCVpp) panels for neutralization breadth testing.

Source	Assay system	Genotypes/subtypes represented	Number of isolates	Reference
Copenhagen hepatitis C program	HCVcc	1a, 1b, 2a, 2b, 3a, 4a, 5a, 6a, and 7a	20	(42, 44, 60, 74)
Johns Hopkins University	HCVcc	1a and 1b	13	(62)
	HCVpp	1a and 1b	113	(25, 67)
University of Nottingham	HCVcc	1, 2, and 3	49	(75)
	HCVpp	1a, 1b, 2, 3, 4, 5, and 6	78	(58, 61, 69)

## Neutralization Sensitivity Varies by HCV Strain, not by Genotype

Heterologous neutralizing activity of HCV-infected human sera is not primarily dictated by the infecting viral genotype. Some studies demonstrated that sera from genotype 1 or 4-infected individuals neutralized genotype 1, 4, 5, and 6 viruses more efficiently than viruses from genotypes 2 and 3 (59, 76). However, these findings may be attributable to strain-specific, rather than genotype-specific effects, since IgG isolated in a different study from serum of subjects infected with either genotype 1, 2, or 3 viruses showed similar neutralization of genotype 1, 2, and 3 HCVpp. There was no relationship between infecting genotype and the genotype of HCVpp that was neutralized by each sample (69). Similarly, in a third study, neutralizing breadth of plasma samples measured using a diverse panel of genotype 1 HCVpp did not differ between individuals infected with genotype 1, 2, or 3 viruses (25). Together, these studies suggest that genetic subtypes of HCV are not neutralization serotypes.

**Figure 2:**
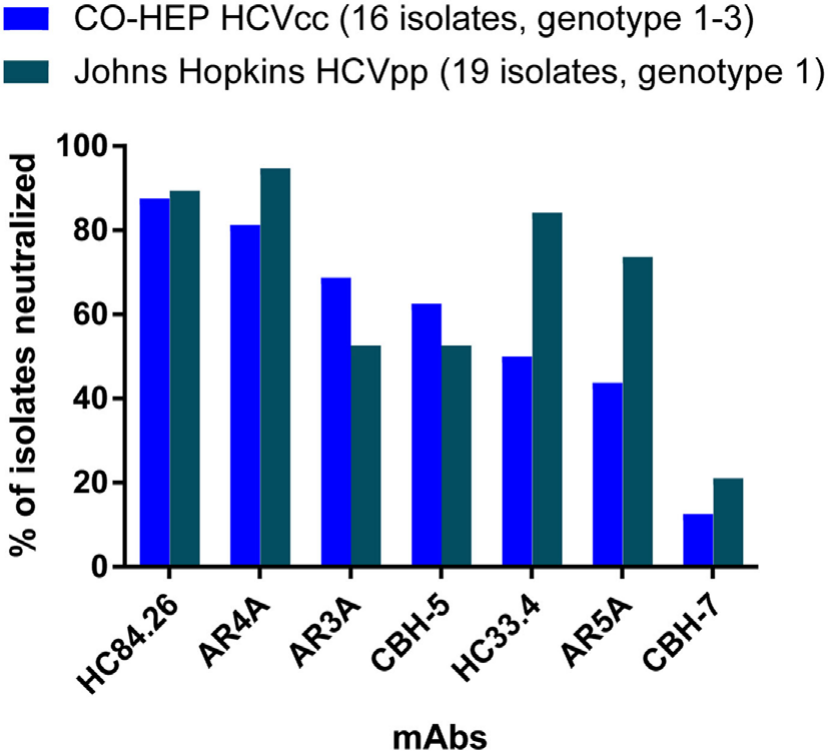
Neutralizing breadth of seven monoclonal antibodies measured using HCV cell culture (HCVcc) and HCV pseudoparticle (HCVpp) panels. Values shown are the percentage of 16 genotype 1–3 HCVcc isolates from the Copenhagen Hepatitis C Program (CO-HEP) neutralized with an IC_50_ less than 10 µg/mL (74), or the percentage of 19 genotype 1 HCVpp isolates from Johns Hopkins University neutralized more than 50% by 10 µg/mL of mAb (72).

Several recent studies have also demonstrated that neutralization sensitivity varies by HCV strain, rather than by genotype. In a study by Carlsen et al., which measured neutralization of 16 HCVcc from genotypes 1–3 by 10 mAbs, 50% inhibitory concentrations (IC_50_) of the same mAb across different HCVcc isolates varied by more than 1,000-fold, with many of the mAbs failing to neutralize all strains in the panel. Notably, this variation in neutralization sensitivity was discernable between isolates from the same subtype as well as between isolates from different genotypes (74). Another study by Urbanowicz et al. measured neutralization of more than 70 HCVpp from genotypes 1–6 by 5 monoclonal antibodies, and also observed wide variation in neutralization sensitivity (61). Again, this neutralization sensitivity varied widely across isolates within each of the subtypes tested. Notably, neutralizing breadth of 7 mAbs was similar when measured in 2 independent studies using either 16 genotype 1–3 HCVcc from the Copenhagen Hepatitis C Program panel (74) or the Johns Hopkins University panel of 19 genotype 1 HCVpp (72), further supporting the concept that neutralization sensitivity to many bNAbs varies by HCV strain, rather than by genotype (Figure 2). Together, these studies suggest that it may be most important for neutralization panels to include many diverse E1E2 strains, even if they represent a single genotype, rather than fewer strains from multiple genotypes.

## Resistance is Conferred by Polymorphisms within and Distant from BNAB Epitopes

Studies using genetically diverse HCVpp and HCVcc have identified multiple mechanisms of HCV resistance to bNAbs. Polymorphisms have been identified in primary HCV isolates that confer resistance to many individual bNAbs and groups of bNAbs. Some bNAb resistance mutations fall within identified bNAb epitopes. When given monotherapy with the monoclonal bNAb designated HCV1, HCV-infected liver transplant recipients developed neutralization resistance mutations at polyprotein positions 415 and 417, within the HCV1 binding epitope (77). Similarly, a naturally occurring F442I polymorphism within the binding epitope of another bNAb, designated HC84.26, confers resistance to neutralization by that bNAb (72). Notably, however, polymorphisms distant from bNAb epitopes can also confer resistance to neutralization. Several studies have demonstrated no association between the level of bNAb resistance of natural HCV strains and mutations within the epitopes of those bNAbs (25, 72). Other studies have identified few mutations in bNAb epitopes in HCV-infected individuals (78), even though bNAbs commonly develop during chronic infection. This observation may be explained in part by the discovery that mutations outside of bNAb epitopes are capable of conferring bNAb resistance (67, 72, 79). Mutations in the central beta sheet of E2, distant from known binding epitopes, can confer resistance to antibodies binding at the CD81 binding site of E2 (72, 79), and a mutation in HVR1 that modulates E2-scavenger receptor-B1 interaction confers resistance to bNAbs AR4A and HC33.4, even if their binding epitopes are fully conserved (67). Prentoe et al. also demonstrate the role of HVR1 in modulating sensitivity of different HCV strains to bNAbs targeting conserved epitopes (80). Although bNAbs in the study did not bind to HVR1, deletion of HVR1 reduced differences in bNAb sensitivity between many HCV strains. Together, these studies suggest that neutralizing breadth of an antibody cannot be predicted solely from the level of conservation of the binding epitope of that antibody.

## Accurate Assessment of Neutralizing Breadth Informs Vaccine Development

Given the extraordinary genetic and phenotypic diversity of HCV, accurate measurement of antibody neutralizing breadth is critical to guide vaccine development. Some studies have suggested that very high titers of bNAbs, as measured using *in vitro* neutralization assays, may be necessary for protection in *in vivo*. Bukh et al. demonstrated that IgG with high cross-neutralizing titers *in vitro* protected chimpanzees against homologous, but not heterologous challenge with HCVcc expressing E1E2 strains identical to those used for *in vitro* testing (28). In addition, measurement of neutralizing breadth of bNAbs using diverse HCVpp and HCVcc panels has shown that no bNAb or serum sample identified to date potently neutralizing all HCV strains (25, 61, 72, 74). These studies suggest that vaccine induction of bNAbs targeting a single HCV epitope may be insufficient for protection. Importantly, neutralizing breadth of bNAb combinations can exceed that of individual bNAbs. In one study, multiple bNAbs, including AR3A and HC84.26, displayed enhanced neutralization against multiple HCV strains when combined with a second bNAb, designated AR4A (74). In another study, bNAbs targeting distinct epitopes displayed enhanced neutralizing breadth when used in combination (73). Two NAbs described in the Mankowski et al. study, designated HEPC74 and HEPC98, displayed both enhanced neutralizing breadth and enhanced potency (synergy) when used in combination. HEPC74 binds at the CD81 binding site of E2 and acts primarily by blocking E2 binding to the CD81 cell surface receptor. In contrast, HEPC98 binds to HVR1 of E2 and acts primarily by blocking E2 binding to the co-receptor SR-B1. Together, these studies suggest that vaccine induction of multiple bNAbs targeting distinct epitopes may be desirable.

## Future Directions

Diverse panels of HCVpp and HCVcc have been used to identify bNAbs, to define neutralizing breadth of antibodies in serum, and to identify mechanisms of HCV resistance to antibody neutralization. However, methods used to define neutralizing breadth can be improved. The vast genetic diversity of HCV is well-described, but the phenotypic diversity of the virus as it relates to neutralization sensitivity remains incompletely understood. Recently, larger and more diverse panels of both HCVpp and HCVcc have been described to better represent the diversity of circulating HCV strains. Currently, available genotype 1 panels encompass most commonly occurring polymorphisms of genotype 1 that isolates worldwide, but fewer isolates are available from genotypes 2 through 7. The expansion of HCVpp and HCVcc panels has identified natural E1E2 strains with resistance to even the most broadly neutralizing mAbs. It is not known whether further expansion of HCVpp and HCVcc panels will identify additional resistant clones, or if strains currently in use accurately represent worldwide phenotypic diversity. Given this limited understanding of the relationship between genetic and phenotypic differences between E1E2 strains, comparison of antibody responses induced by different vaccines has been hampered by the use of different neutralization panels in different laboratories. Therefore, future goals for the field should include optimization and standardization of a genotype 1 neutralization panel representing the many diverse E1E2 isolates already available, along with expansion and standardization of neutralization panels encompassing other genotypes. These standardized panels could be used to accurately evaluate and compare neutralizing antibodies and post-vaccination immune sera tested in different laboratories, accelerating development of an HCV vaccine.

## Author Contributions

VK wrote the manuscript. JB edited the manuscript and prepared figures.

## Conflict of Interest Statement

The authors declare that the research was conducted in the absence of any commercial or financial relationships that could be construed as a potential conflict of interest. The handling Editor declared a past co-authorship with the authors.
